# *Snca-*GFP Knock-In Mice Allows Tracking the Endogenous α-Synuclein in Action

**DOI:** 10.1523/ENEURO.0544-20.2021

**Published:** 2021-01-21

**Authors:** Safak Er

**Affiliations:** HiLIFE, University of Helsinki, Helsinki, 00014, Finland

## Abstract

**Highlighted Research Paper:** *Snca*-GFP Knock-In Mice Reflect Patterns of Endogenous Expression and Pathologic Seeding, by Anna Caputo, Yuling Liang, Tobias D. Raabe, Angela Lo, Mian Horvath, Bin Zhang, Hannah J. Brown, Anna Stieber, and Kelvin C. Luk

α-Synuclein (aSyn) is one of the most abundant proteins in the brain. It is highly expressed in neurons, and it participates in the regulation of presynaptic vesicle fusion and trafficking. However, it was neither its abundance nor its physiological role that placed aSyn under the spotlights of neurodegeneration research. Interest in the protein started after back-to-back discoveries, specifically: hereditary mutations in aSyn encoding gene (*SNCA*) causing Parkinson’s disease (PD) and presence of filamentous aSyn in Lewy bodies (Goedert and Spillantini, 1998).

Lewy bodies are proteinaceous, neuronal inclusions observed in the postmortem brains of patients with PD and other neurodegenerative diseases collectively referred as synucleinopathies. These diseases show differences in the brain area or cell type where aSyn pathology and cellular loss are more pronounced (Goedert and Spillantini, 1998). However, unanswered questions about synucleinopathies are similar: (1) How progressive neurodegeneration is related to aSyn pathology? and (2) What is the most detrimental form of accumulated aSyn?

Presence of Lewy bodies in both familial and sporadic PD cases supports the hypothesis that aggregation of wild-type (wt) aSyn is sufficient for disease pathology. Although certain genetic mutations can cause synucleinopathies, sporadic cases are by far the most common. However, most transgenic aSyn animal models are based on overexpression of wt or mutated aSyn, often controlled by a non-native promoter. Thus, the data obtained using these models has limited transferability for idiopathic cases of synucleinopathies and physiological behavior of aSyn. These models also have short comings in recapitulation of prion-like behavior of aSyn (Caputo et al., 2020).

Initial proof for prion-like spreading of aSyn pathology was provided by analyzing postmortem tissue from PD patients with no familial history of the disease. Aggregated aSyn was also observed in neurons transplanted to the brains of PD patients (Dehay et al., 2016). Later, initiation of Lewy body-like inclusions and spreading aSyn pathology were successfully recapitulated in wt rodent brains by intracranial injection of exogenous aSyn fibrils, specifically, recombinant aSyn preformed fibrils (PFFs) and aSyn aggregates derived from human brains (Luk et al., 2012; Masuda-Suzukake et al., 2013).

Previously established aSyn models have cultivated our knowledge on its normal function and pathologic accumulation. However, there are still unmet needs in the field for understanding how endogenous aSyn behaves in healthy brain and during the disease. Plus, live tracking of the physiological protein would be beneficial for comparing tissue/cell types with different expression levels of aSyn and how they respond to aggregation initiators like exogenous aSyn.

The recent *eNeuro* article by Caputo et al. (2020) has presented a novel knock-in (KI) mouse with a GFP tag at the C terminus of endogenous aSyn. This well-characterized model offers tracking of natively expressed aSyn in action. The expression of aSyn-GFP fusion protein is shown to be at the same level with wt aSyn, which is expected since fusion protein is expressed in presence of native regulatory elements. Neither ectopic protein expression, nor unexpected pathologic post-translational modifications are reported. It is worth mentioning that the authors also validated the presence of aSyn-GFP in the peripheral cells (e.g., red blood cells) that normally express aSyn. Distribution of aSyn-GFP almost completely matches the distribution of aSyn in wt mouse; outside CNS, in the brain and spinal cord, and, most importantly, at presynaptic regions.

The fusion protein maintained its physiological location and participated in synaptic vesicle formation together with Synapsin 1. When simulated with KCl, aSyn-GFP showed similar kinetics compared with wt protein. As discussed by authors, it is clear that GFP tag interferes with aSyn biology to some extent. Exogenous fibril formation with monomers of GFP-tagged aSyn protein had slower kinetics. However, resulting aSyn-GFP PFFs were still functional for initiating aSyn aggregation. Also, the fibril formation kinetics improved with the addition of wt monomers during assembly.

Interference of the GFP tag had its most significant effects on PFF-induced aSyn pathology in the brain of KI mice. Compared with wt mice and heterozygous littermates, homozygous KI mice accumulated very low numbers of PFF-induced neuronal aSyn aggregates. Similarly, exposure with PFFs induced few aSyn aggregates in primary cultured neurons from these mice. Despite lower incidence of pathology, aSyn-GFP was incorporated within phosphorylated aSyn inclusions, and, similar to aSyn pathology in patients’ brains, aggregates were detergent insoluble.

Experiments with wt PFFs and KI mice once again highlight the importance of endogenous aSyn in pathology formation (Luk et al., 2012). Previously, Osterberg et al. (2015) have used PFFs, synergistically with mice overexpressing human aSyn-GFP and monitored formation of Lewy-body like aSyn inclusions via *in vivo* imaging. Their results demonstrated that recruitment of endogenous aSyn-GFP by exogenous fibrils is necessary for aSyn pathology. Likewise, Caputo et al. (2020) used their novel model for live imaging and captured endogenously expressed aSyn in action (see Movie 1). Live cell imaging of primary hippocampal neurons cultured from heterozygous KI mice shows that after PFF inoculation, aSyn-GFP acquired “serpentine like” structures and behaved divergently than physiological aSyn. The movie also shows integration of aSyn-GFP to a larger aSyn accumulate. It is impressive to easily observe multiple behaviors of one protein, just in few seconds. Further studies can be conducted with various tissues of heterozygous KI mice for investigating aSyn biology in real time, with or without additional fluorescence trackers.

Movie 1. Live imaging of PFF-transduced *Snca^wt/GFP^* neurons. *Snca^wt/GFP^* neurons plated on MatTek were transduced at 10 Days *In Vitro* with 2 μg/ml of mouse wt PFF and image, 5 d posttransduction, for the indicated time every 15 min at 37°C, in the presence of 5% CO_2_. Arrows indicate vesicular aSyn-GFP and arrowheads serpentine-like or aggregated aSyn-GFP. Scale bar: 10 μm. (From Caputo et al., 2020.) 10.1523/ENEURO.0544-20.2021.video.1

The limitation of the model is the lower incidences of PFF-induced aSyn pathology, which is slightly ameliorated in heterozygous mice. Nevertheless, *Snca-*GFP KI mice provide a unique, well-characterized tool for connecting the dots between physiological and disease-related behavior of endogenous aSyn. KI mice show no defects in development or at later stages, except minimal disruption in physiological aSyn function that does not affect behavior of the animals. In addition to the possibility of dynamic monitoring of aSyn in CNS neurons, same features can be employed outside of the brain tissue where aSyn is also expressed, but less studied. This new model will surely help to answer important questions about aSyn physiology and synucleinopathies, and intriguingly, generate new ones along the way.

## Synthesis

Reviewing Editor: Christophe Bernard, INSERM & Institut de Neurosciences des Systèmes
